# Chronic Leptospirosis in a Breeding Bull: A Case Report

**DOI:** 10.3390/microorganisms13071695

**Published:** 2025-07-18

**Authors:** Gabrita De Zan, Antonio Carminato, Monia Cocchi, Giacomo Catarin, Irene Pascuci, Laura Lucchese, Laura Bellinati, Letizia Ceglie, Elisa Mazzotta, Mario D’Incau, Martina Ustulin, Laura Grassi, Alda Natale

**Affiliations:** 1 Istituto Zooprofilattico Sperimentale delle Venezie (IZSVe), Viale dell’Università 10, 35020 Legnaro, PD, Italymcocchi@izsvenezie.it (M.C.); llucchese@izsvenezie.it (L.L.); lbellinati@izsvenezie.it (L.B.); emazzotta@izsvenezie.it (E.M.); anatale@izsvenezie.it (A.N.); 2 Centro Tori Moruzzo, Strada dei Quattro Venti SP83 7, 33030 Moruzzo, UD, Italy; 3 Istituto Zooprofilattico Sperimentale della Lombardia e dell’Emilia-Romagna (IZSLER) “Bruno Ubertini”, Via Bianchi 9, 25124 Brescia, BS, Italy; mario.dincau@izsler.it

**Keywords:** cattle, *Leptospira*, leptospirosis, bovine genital leptospirosis, One Health

## Abstract

Leptospirosis is a (re-)emerging and global zoonotic disease. Given the complex host-pathogen interaction and the numerous environmental risk factors related to the transmission, a One Health approach to both disease prevention and control is needed. Occurring at the human–cattle–environment interfaces, bovine leptospirosis represents a zoonotic risk for the professionals in the field, besides being a potential cause of significant economic losses due to the bovine reproductive disorders. Although climatic change is a potential factor in exacerbating the risk of leptospirosis in Europe, this disease remains largely neglected, with several knowledge gaps in research, investigations, and diagnosis of bovine genital leptospirosis syndrome across the continent. The present report describes the results of the diagnostic investigations on a case of chronic bovine leptospirosis in a breeding bull. Following the seroconversion to *Leptospira* Sejroe var Hardjo after the arrival of the animal in a quarantine facility, a monitoring plan including both serological/molecular analyses and a therapeutic protocol was undertaken. The bull exhibited a persistent seroconversion and a repeated positivity for *Leptospira* to real-time PCR in urine samples, indicative of a chronic shedder pattern. This report emphasizes the diagnostic and management challenges in the context of such a complex but frequently overlooked disease.

## 1. Introduction

Leptospirosis is a worldwide-distributed bacterial zoonotic disease [1]. Affecting over 1 million humans with 58,900 estimated deaths per year, leptospirosis is among the leading zoonotic causes of morbidity and mortality [1]. Notwithstanding, leptospirosis sits globally in a “vicious circle of neglect”, as confirmed in a recent review that included leptospirosis in the list of the 26 priority neglected zoonotic diseases [2,3].

The species of *Leptospira* cluster into three groups: pathogens, non-pathogens, and biochemical intermediates. Historical classification classifies *Leptospira* species into serovars based on serotyping methods [4].

Leptospirosis infection results from a direct or indirect exposure, this latter related to the environment contaminated by carrier animals [4]. Rodents are generally considered the main reservoirs of this infection; however, the epidemiological cycle of leptospires depends on the specific serovar/genospecies involved, and in rural environments, other species have been identified as carriers, including cattle, sheep, pig, horse, and dog, as well as wild animals [5,6]. Common portals of entry are mucous membranes and open wounds/abrasions. Leptospires are able to spread and cause systemic and fatal disease thanks to their ability to escape the immune system, their motility capacity, and their resistance to complement proteins [4].

In Europe, leptospirosis is (re-)emerging in both humans and animals and is likely to be favoured by global and climate change [5,7,8].

The zoonotic aspects of bovine leptospirosis are under-resourced and largely neglected in Europe, despite the fact that several countries reported relatively high seroprevalence in cattle [9]. Specifically, leptospirosis registers a knowledge gap on the risk of transmission through semen by artificial insemination [9]. Although the high prevalence of bovine genital leptospirosis (BGL) in Brazil has led to its recognition as a specific syndrome, no studies on BGL have been conducted across Europe in the last 20 years [9,10]. In addition, the European Union Animal Health Law no longer includes leptospirosis among the notifiable diseases [11].

The risk is multifactorial and likely related to the biosecurity measures, to the *Leptospira* serovar/genospecies involved, and to the environmental conditions even if the role of rodents as a source of infection in cattle is poorly investigated [9].

Cattle are the maintenance host for serovar Hardjo (types Hardjobovis and Hardjoprajitno, serogroup Sejroe) [9]. The maintenance of the serovar Hardjo is exclusively contingent upon host factors, whereas infections attributed to other serovars/serogroups are regarded as incidental [12,13]. While no successful isolation of *Leptospira* strains from naturally infected cattle was registered in Europe between 2001 and 2021 [9], a recent study conducted in Austria in dairy and beef cattle has successfully cultured three isolates identified as *Leptospira* (L.) *borgpetersenii* serogroup Sejroe serovar Hardjobovis, cgMLST cluster 40 [14]. *L. borgpetersenii* serovar Hardjo ST 152 has also been isolated in the context of 5 independent outbreaks of hypofertility and abortion in dairy cattle in Italy (personal communication, MD).

The manifestation of leptospiral infection in cattle displays a wide range of symptoms, mainly depending on the age of the animals. Uncommon manifestations of severe and acute disease in young animals are associated with serogroups Pomona, Icterohaemorrhagiae, and Grippotyphosa [12]. The chronic form caused by serovar Hardjo typically affects adults, with a slightly different manifestation between dairy and beef cattle [12]. Specifically, in order of frequency, the chronic *Leptospira* infection in cattle causes abortion (58.6%), fertility disorders, and reduction in milk yield. The acute infection could manifest with a sudden drop in milk production, hyperthermia, haemoglobinuria, and icterus [9,15]. In bulls, leptospirosis is underestimated, and consequently, poorly reported. Both the clinical manifestation and the impact on the semen quality and fertility remain unclear, despite the description of cases of genital infection and the recognition of leptospirosis as a disease potentially transmitted by coitus or semen [16,17].

Leptospirosis can potentially cause significant economic losses due to the reduced livestock productivity related not only to the reproductive disorders and compromised reproductive performance but also to the rise of total costs and to the rate of carryover cows [9,18,19]. In a recent study conducted on Jersey dairy cows in Brazil, the losses from reduced productive and reproductive performance caused by leptospirosis were determined to be around 84% of the annual gross margin [19].

It is essential to establish appropriate and harmonised protocols for the correlation between serogroups and clinical disease in cattle. Local studies are a valuable source of information to understand the epidemiology of the disease and thus to develop prevention and control measures [9,20,21].

The aim of the present study is to provide a detailed description and a comprehensive documentation of the diagnostic findings in a case of chronic leptospirosis in a breeding bull. This field, especially in Europe, remains largely underinvestigated due to the lack of longitudinal monitoring plans including the trend of results over time.

## 2. Case Presentation

A 2-year-old Simmental bull imported from Ireland was admitted and individually housed in a quarantine facility in Italy (C2FF+6F Tolmezzo). Before being transferred to a genetic center to initiate the semen collection, the animal underwent the routine diagnostic screening.

**Figure 1:**
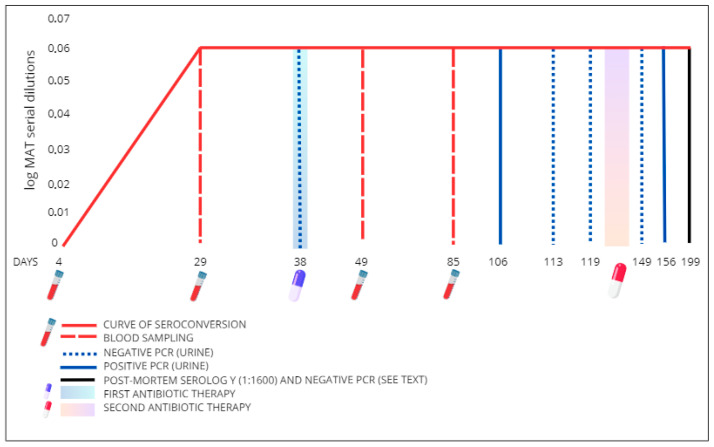
Serological monitoring plan, PCR screening plan and antibiotic therapies. First antibiotic therapy: intramuscular administration of oxytetracycline cloridate (Oxtra Mv 10, FATRo S.p.A., Ozzano dell’Emilia (BO), Italy—3 days). Second antibiotic therapy: subcutaneous administration of ceftiofur (Ceftionil, Virbac S.r.l., Milano (MI), Italy—6 days). The MAT was performed on 4 serum samples collected on days 4, 29, 49, and 85.

The protocol included the examination of specific antibodies against *Leptospira* spp. through the microagglutination technique (MAT) [22]. The antigen panel included the following serogroup/serovars distributed by the Italian Reference Center for Animal Leptospirosis: Australis/Bratislava, Canicola/Canicola, Grippotyphosa/Grippotyphosa, Icterohaemorrhagiae/Copenagheni, Icterohaemorrhagiae/Icterohaemorrhagiae, Pomona/Pomona, Tarassovi/Tarassovi, Ballum/Ballum, and Sejroe/Hardjo. A cut-off value equal to or greater than 1:100 was considered positive. Given the seroconversion against the latter with a titer of 1:1600 at the second sampling (day 29), the animal was subjected to a serological monitoring plan and a molecular screening on urine combined with a therapeutic protocol (Figure 1).

Molecular analysis for *Leptospira* detection was performed on six urine samples collected by spontaneous urination from the animal on days 38, 106, 113, 119, 149, and 156. For each sample, 2 mL of urine was centrifuged at 12,000× *g* for 20 min at 4 °C, and the pellet was re-suspended in 0.2 mL of sterile PBS. To enhance nucleic acid recovery, we added 20 µg of poly-A carrier (Sigma-Aldrich, St. Louis, MO, USA). A pre-lysis step preceded DNA extraction, with 100 µL of re-suspended pellet incubated with 2.5 µL of lysozyme (10 mg/mL in 10 mM Tris-HCl, pH 8.0, Sigma-Aldrich, St. Louis, MO, USA) at 37 °C for 15 min. DNA was then extracted using the ID Gene^®^ Mag Universal Extraction Kit (IDvet, Grabels, France) on the KingFisher™Flex Purification System (Life Technologies, Carlsbad, CA, USA), following the manufacturer’s instructions.

Total DNA was analysed using a real-time PCR (rPCR) protocol [23], targeting an 87-bp genomic fragment within the *16S rRNA* gene specific to pathogenic *Leptospira* species [24] (Table 1). Samples were classified as positive when cycle threshold (Ct) values were <38, doubtful in case Ct ranged between 38 and 40, and negative with Ct values ≥ 40 or lacking a FAM fluorescence signal.

During the monitoring plan the MAT serological positivity to *L.* Sejroe var Hardjo persisted, and leptospiral DNA was detected in urine samples both on day 106 (Ct of 37.70) and on day 156 (Ct 36.50), despite the applied therapies (Figure 1). Based on these results, albeit there were no evident clinical signs, the bull was humanely euthanized.

Due to biosecurity measures, it was not possible to perform a complete necropsy in the field. Since urinary bladder was empty, the urine was not available for analyses. Only blood (i.e., coagulum), semen (frozen and sent to the Istituto Zooprofilattico Sperimentale della Lombardia e dell’Emilia-Romagna), liver, kidney, and testicle (refrigerated tissues) were collected for further diagnostic investigations (day 199). Serology was performed on the coagulum, while rPCR, as previously described, was performed on liver, kidney, and testicle tissue samples.

Two millilitres of the semen sample was centrifuged at 13,000× *g* for 30 min at 4 °C, the supernatant discarded, and the pellet incubated with 50 µL of lysozyme (10 mg/mL in 10 mM Tris-HCl, pH 8.0, Sigma-Aldrich, St. Louis, MO, USA) at 37 °C for 30 min. DNA was then extracted using the PureLink™ Genomic DNA Mini Kit (Invitrogen^®^, Waltham, MA, USA), following the manufacturer’s instructions. The extracted DNA was amplified using a real-time multiplex PCR protocol targeting a *16S rRNA* gene fragment [25] to detect pathogens from the *Leptospira* genus and a *lipL32* gene fragment to identify pathogenic *Leptospira* species [26] (Table 1). The assay was performed using the QuantiFast Pathogen PCR +IC Kit (Qiagen, Hilden, Germany) on a CFX96 Touch Real Time (Bio-Rad, Hercules, CA, USA) instrument under the following thermal cycling conditions: initial denaturation at 95 °C for 5 min, followed by 45 cycles at 95 °C for 15 s and 60 °C for 30 s. Samples were considered positive for the *Leptospira* genus if the *16S rRNA* gene exhibited Ct values between 5 and 38, while *lipL32* positivity, indicating the presence of a pathogenic *Leptospira* species, was considered for Ct values between 5 and 40. The co-extraction and co-amplification of an exogenous internal control (Internal Control—High Concentration, Qiagen, Hilden, Germany) validated each negative result, ensuring the absence of inhibition throughout the process [25,26].

Serology at the time of culling (day 199) confirmed seroconversion to *Leptospira* Sejroe serovar Hardjo with a MAT titre of 1:1600, while all the other samples tested negative to PCR.

Attempts to isolate the bacterium from the urine collected during quarantine (day 38) and from the liver, kidney, and testicle sampled at the time of culling (day 199), using EMJH (Ellinghausen–McCullough–Johnson–Harris) medium [22], were unsuccessful.

Although both urine samples tested weakly positive by rPCR (Ct 36.50 and 37.70), an attempt was made to genotype the *Leptospira* species using the 7-loci multilocus sequence typing scheme proposed by Boonsilp et al. in 2013 [27], which targets the housekeeping genes *glmU*, *pntA*, *sucA*, *tpiA*, *pfkB*, *mreA*, and *caiB*, as previously described [28]. Unfortunately, amplification was unsuccessful due to the low bacterial DNA concentration.

**Figure 2:**
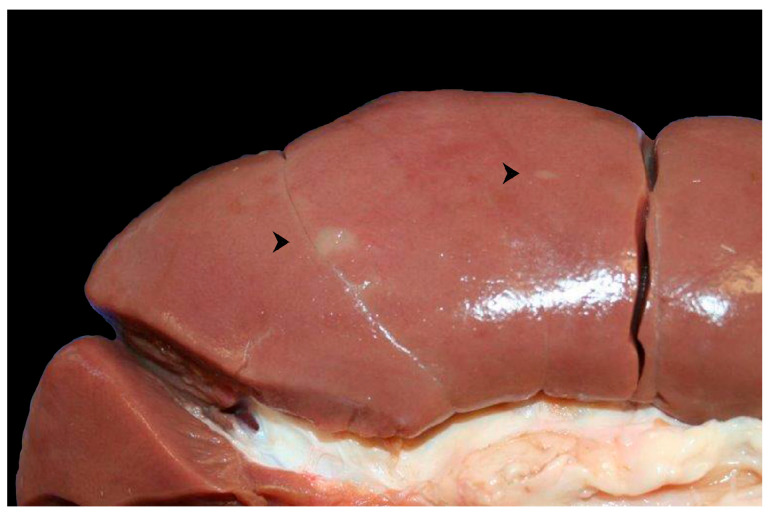
Kidney, rare, multifocal, irregularly round, grey lesions on the surface (arrowheads).

Macroscopically, the kidney presented rare, randomly distributed, irregularly shaped (maximum dimension of about 0.5 X 1 cm), flat and grey lesions with irregular and indistinct borders (Figure 2). On the cut surface, these lesions partially extended to and obscured the cortex. No significant lesions were observed in the liver or in the testicles.

Samples from kidney, liver, and testicles were formalin-fixed and paraffin-embedded (FFPE) for histopathological examination.

**Figure 3:**
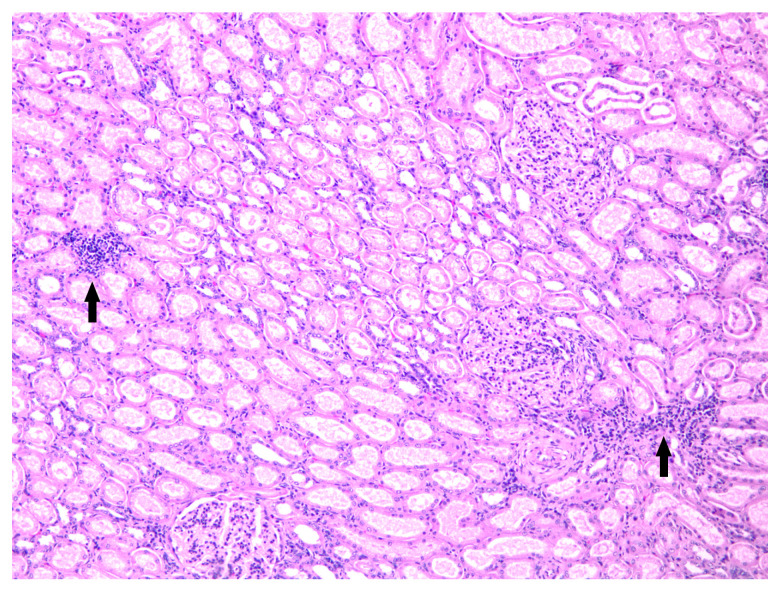
Kidney, scattered foci of chronic interstitial nephritis (arrows) mainly composed of lymphocytes and plasma cells (hematoxylin and eosin, 10×).

Microscopically, the kidney showed a mild and multifocal interstitial nephritis characterized by scattered inflammatory foci mainly composed of lymphocytes and plasma cells together with rare areas of mild peritubular/interstitial fibroplasia (Figure 3).

**Figure 4:**
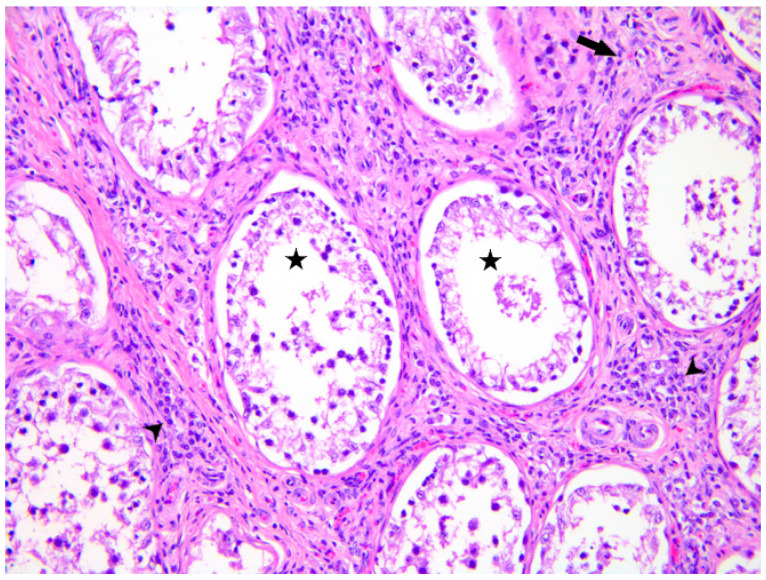
Testicle, mild multifocal chronic interstitial orchitis (arrowheads) composed of histiocytes, lymphocytes and fewer plasma cells and neutrophils with severe diffuse seminal atrophy (asterisks) and mild peritubular/interstitial fibroplasia (arrow) (hematoxylin and eosin, 20×).

The liver exhibited scattered foci of periportal lymphoplasmacellular infiltration. The testicle revealed moderate diffuse peritubular/interstitial fibroplasia associated with mild to moderate and multifocal chronic interstitial inflammation composed of histiocytes, lymphocytes, and fewer plasma cells and neutrophils. The seminiferous tubules showed diffuse and severe reduction in germ cells (spermatids/spermatocytes) and prominence of Sertoli cells. The testicular histopathological features were consistent with chronic interstitial orchitis with severe seminal atrophy (Figure 4).

The main purpose for performing histochemical and immunohistochemical investigations was to identify the presence of leptospires in the histological sections. Histochemical silver stain was performed using the Warthin-Starry kit commercialized by Diapath S.p.A. (Martinengo (BG), Italy). Automated immunohistochemistry (Ventana Discovery Ultra, Roche, Basel, Switzerland) was performed using a primary polyclonal rabbit antibody against *L. interrogans* serovar Hardjo type Prajitno, strain Hardjoprajitno, supplied by the Amsterdam UMC Leptospirosis Reference Centre at a dilution of 1:500. Briefly, 3-μm-thick FFPE tissue sections were mounted on adhesive glass slides, deparaffinized in aqueous-based detergent solution (EZ Prep, Ventana, Roche, Basel, Switzerland) and subjected to antigen retrieval using a pH 6 citrate buffer. The detection of reaction was obtained by using a secondary anti-rabbit HRP antibody (DISCOVERY OmniMap anti-Rb HRP (RUO), Ventana, Roche, Basel, Switzerland) and the DAB chromogen (DISCOVERY ChromoMap DAB Kit (RUO), Ventana, Roche, Basel, Switzerland). Sections were counterstained with Mayer hematoxylin solution, dehydrated, and mounted (Eukitt, Kaltek, Padova (PD), Italy). Positive controls were prepared by applying a thin layer of a suspension of *L. interrogans* culture between two sections of formalin-fixed bovine liver in order to obtain a sort of “tissue-sandwich”. The obtained preparation underwent formalin fixation for 24 h before processing [29]. The positive controls highlighted strong positivity of the spirochetes within the “tissue-sandwich”, whereas all the analysed samples of the bull were negative at both silver stain and anti-*Leptospira* immunohistochemistry.

Finally, semen quality analysis (i.e., microscopy and motility test on thawed semen) revealed low semen concentration and motility (personal communication, GC).

## 3. Discussion

Despite being a zoonotic disease that poses a significant ongoing public health threat worldwide, leptospirosis still represents a complex disease with several knowledge gaps and difficulties in both diagnosis and management. On one hand, leptospirosis shows a confirmed global trend of re-emergence, both in humans and in animals, with an increasing incidence even in mild climate regions, including Europe, likely due to climate change [1,5,7,8]. On the other hand, it remains widely neglected from both a legislative and sanitary perspective [11], which is quite surprising considering it has recently been included in the list of the 26 priority neglected zoonotic diseases [2,3].

In this context, leptospirosis in livestock, and more specifically in bovines, is even more insidious as several factors converge in this field. Cattle are the primary reservoir for serovar Hardjo. However, they can potentially be susceptible also to other species and serovars [12]. In New Zealand, cattle have been identified as potential spillover hosts for serovar Ballum, although knowledge about this species is limited [30]. During an outbreak of congenital jaundice in aborted bovine foetuses in Belgium, the incidental infection with non-maintenance serovars, such as Grippotyphosa and Australis, which were the most prevalent serogroups, was reported [20,31]. *L. kirschneri* and *L. interrogans* serogroup Pomona and serogroups Icterohaemorrhagiae and Australis have also been associated with reproductive diseases [12,13,31,32].

Despite its high seroprevalence in cattle across Europe, the role of *Leptospira* within the One Health framework remains insufficiently investigated, with an almost complete lack of data on the zoonotic risk associated with artificial insemination [9]. In New Zealand, accidental human infection with serovars endemic in livestock is common. Moreover, shedding of non-vaccine serovars has also been reported; therefore, this occupational zoonosis continues to be a risk, regardless of the vaccination status of the herd [33].

The risk factors for bovine leptospirosis are numerous and complex, encompassing a range of ecological, managerial, and individual factors. These include environmental factors, herd management practices, biosecurity measures, the clinical condition of the animal, and the presence of infectious comorbidities. Furthermore, although not subjected to statistical analysis, the presence of rodents may also represent a potential risk factor, as observed in other species [9]. In the present case, it was not possible to identify with certainty the potential source of exposure for the bull. The incubation period for leptospirosis ranges from 2 to 30 days, with an average of 7–10 days, and seroconversion usually takes place 10–15 days after infection [5,12]. Therefore, the absence of antibodies 4 days after the arrival of the animal at the genetic centre suggests that exposure to *Leptospira* may have occurred before or during transport or in the quarantine facility. The strictly controlled environment of the quarantine facility makes the exposure after arrival unlikely, although it cannot be completely ruled out. It is noteworthy that during the same period, all the other bulls housed in the same quarantine facility tested negative for *Leptospira* using MAT.

The main manifestations of BGL include embryonic death, abortion, stillbirth, premature birth, reproductive failure, such as oestrus repetition and subfertility, and milk drop syndrome [9,10,15]. The infection of the genital tract by leptospires has been traditionally considered secondary to the bacteraemia after the phase of renal infection. Conversely, a review of the literature reveals that there is a growing body of evidence that points towards the definition of BGL as a specific syndrome not associated with the well-known renal/systemic disease [9,10,16]. Nevertheless, further research on reproductive disorders associated with leptospiral infections in ruminants is recommended, given that the pathogenesis of this insidious chronic infection remains poorly defined and data are scarce [34]. A postulated hypothesis regards the reduction, compromised secretion, or dysregulation of the composition of the histotroph [16]. Moreover, experimentally, exposure of bovine endometrial epithelial cells or human monocytes to heat-killed *Leptospira* or *Leptospira* outer membrane did not induce cytokine production. Therefore, it may be assumed that leptospires are not recognized by the immune system in the uterus, thus allowing the progression of the infection that causes BGL [35].

Concerning our case, in which the bull was asymptomatic, the available literature provides very little information. The clinical signs of BGL in bulls are not well documented, although the identification of leptospires in semen has been reported [16]. For this reason, a precautionary approach that includes the addition of antimicrobials in semen and in media for embryo culture is recommended as a means of controlling this syndrome [16].

Furthermore, the diagnostic work-up and the management of BGL imply a comprehensive and intricate process that needs specific and detailed protocols (e.g., Herdsure^®^ protocol—Leptospirosis). In addition to the systematic testing and quarantine of newly introduced animals, the diagnostic process includes an initial step aimed at identifying carriers through serological screening of the herd using the MAT method, in addition to molecular screening on samples from the reproductive system (e.g., mucus, uterine mucosa, and semen). This latter approach specifically focused on animals with signs of reproductive disorders [16]. The subsequent monitoring of carriers is conducted through a three-step method that includes the antibiotic treatment, the control and management of the environment, and the monitoring of reproduction and herd vaccination plans [16]. It is notable that, similarly to our case, the persistence of high serum titres has been documented in cows inoculated with *L.* Hardjo [36].

The diagnosis of leptospirosis is inherently complex, and even more so in bovine cases given the lack of harmonisation in diagnostic protocols, as evidenced in the literature [9,22]. Tests for leptospirosis are classified into two categories, depending on the target: detection of leptospires versus detection of anti-leptospiral antibodies. Several factors may influence the choice of an assay (e.g., availability of resources or expertise, time, purposes) [9,22,37]. Moreover, in the context of BGL, the tests on kidney, urine, or genital tract from asymptomatic subjects are indicative of a state of chronic carrier, but not necessarily of the presence of the disease [22]. Given that no single assay is optimal (i.e., bacterial culture, molecular analysis, or serology), a multiple diagnostic approach is recommended (i.e., the use of two techniques in parallel) in order to maximize the diagnostic sensitivity [37,38].

The reference test for the identification of carriers is urine culture, which also allows the genetic and serovar typing that is useful for epidemiological studies, setting of MAT diagnostic panels, and vaccine production. However, the bacterial culture is restricted to reference laboratories, it has a low sensitivity, and it is time-consuming (up to 4 months) [22]. Specifically, serovar Hardjo generally displays an extremely low and fastidious growth, it requires an enriched culture medium, and the cell viability is negatively affected by a prolonged exposure to urine (critical maximum time < 2 h) [14,37,39]. The failure of several attempts at culture, as observed in our case, could simply indicate that the number of leptospires was not detectable at the time of testing (e.g., dilution effect of bovine urine) or they were not viable (e.g., antimicrobial therapies applied). However, this does not rule out the possibility that the bull was a chronic shedder [22].

PCR is recognized as a reliable, quick, and precise diagnostic method if compared to traditional techniques, such as microbiological culture and dark field microscopy, but it requires experienced staff and a strict quality control process, as well as different kinds of sample processing, depending on the matrix and species tested [22,40]. The advantages of molecular investigations include a higher sensitivity and the possibility of DNA quantification and sequencing for species identification [37,40]. However, in infected animals leptospiruria can be intermittent, as possibly observed in our case, and leptospires can still be isolated from the kidney even after leptospiruria is apparently finished [41,42]. Moreover, in our case the potential impact of bovine urine dilution as well as the antibiotic therapies could also have affected PCR sensitivity [22]. The negative PCR results obtained from the other tested samples (i.e., kidney, liver, testicle, and semen) may have been due to several factors. These include a low number of leptospires or an uneven distribution of the bacteria within the tested tissues, the influence of the antimicrobial treatments, and the presence of amplification inhibitors associated with tissue autolysis [22]. Furthermore, inconsistency between sperm viability for reproductive purposes and serological and molecular detection of leptospires in this matrix has been reported. This suggests the need to combine methods to determine the carrier status in bulls [21].

The low number of leptospires, the application of therapeutic protocols, and the lower sensitivity of the analytical methods used may have contributed to the negative results obtained with the histochemical and immunohistochemical tests applied [22]. In the recovery stages of the disease and in its subclinical form, the localization of leptospiral microcolonies as intratubular aggregates, rather than in the interstitium, could also affect the ability to identify the agent by histochemical and immunohistochemical techniques [43]. Nevertheless, the pathologic findings, even if neither indicative nor specific, are compatible with the presence of a chronic form of leptospirosis. In our case, the presence of indistinct foci of grey discoloration of renal parenchyma, mainly in the cortex, could be the only residual lesion after acute infection in bovine and is also reported in chronically infected asymptomatic sheep [6,12,43]. Most cases of bovine leptospirosis also show mild and non-specific histologic changes. According to the literature, mild periportal inflammation and foci of lymphoplasmacytic cortical interstitial nephritis, the latter reported to decrease as the lesion regresses, have been observed also in our case [6,12,43].

The available information on genital lesions, immunity response, and consequences on semen quality and fertility related to BGL in bulls is limited, although the semen is a potential transmission route and the presence of leptospires in this matrix has been documented [16]. In a subset of examined bulls (3 out of 203) seroactive (MAT ≥ 400) to serovars from the Sejroe serogroup, Maiolino et al. [21] found the presence of semen alterations such as necrospermia and azoospermia. The direct detection method (PCR) yielded negative results for all these animals. Orchitis and balanoposthitis assessed by physical examination have also been reported as an “atypical manifestation” in a dog infected with serovar Canicola [44]. Despite the presence of chronic orchitis and low semen quality at a young age that were observed also in our case, and given the scarcity of information, it is not possible to draw conclusions about the potential influence of *Leptospira* infection on the testicular tissue and on semen viability. It is evident that these findings give rise to further concerns in the context of BGL, as recently highlighted in literature [16].

## 4. Conclusions

Considering all these aspects, bovine leptospirosis is a complex disease to deal with from an epidemiological, clinical, and diagnostic perspective. Based on the data obtained from the animal under investigation, such as a persistent seroconversion and repeated PCR-positive results, we believe it might have been affected by a chronic form of infection. The bull was supposed to be a chronic shedder for *Leptospira* with intermittent shedding of leptospires, despite the antibiotic therapies and in the absence of any clinical sign. Given the relevant impact of BGL on the reproductive sphere, the chronic, silent, and subclinical manifestation of the syndrome, the zoonotic potential, and the underestimation of the disease, especially in the bull, where the infection remains to be clarified, further investigations should be encouraged to enrich the existing knowledge on this neglected topic. Serological screening on pooled milk (ELISA) in dairy cattle farms is a rapid and efficient method to monitor and control the infection in this species. Surveillance on bulls is also pivotal to better understand and manage the disease and should be performed by serology (MAT) and direct detection (PCR) on semen [16].

## Figures and Tables

**Table 1:** Targets of the rPCR used, oligonucleotide sequences of primers and probes, fragment lengths of the amplified segment, and corresponding references.

Target	Oligonucleotide Sequence (5′-3′)	Fragment Length	Reference
*16S rRNA* gene of pathogenic leptospires	Forward: 5′-CCCGCGTCCGATTAG-3′ Reverse: 5′-TCCATTGTGGCCGRACAC-3′ Probe: 5′-FAM-CTCACCAAGGCGACGATCGGTAGC-TMR-3′	87 bp	[23]
*16S rRNA* gene for *Leptospira* genus	Forward: 5′-TAGTGAACGGGATTAGATAC-3′ Reverse: 5′-GGTCTACTTAATCCGTTAGG-3′ Probe: 5′-Cy5-AATCCACGCCCTAAACGTTGTCTAC-BHQ1-3′	103 bp	[24]
*lipL32* gene of pathogenic leptospires	Forward: 5′-AAGCATTACCGCTTGTGGTG-3′ Reverse: 5′-GAACTCCCATTTCAGCGATT-3′ Probe: 5′-FAM- AAAGCCAGGACAAGCGCCG –BHQ1-3′	242 bp	[25]

## Data Availability

The original contributions presented in this study are included in the article. Further inquiries can be directed to the corresponding author.

## References

[1] Costa F., Hagan J.E., Calcagno J., Kane M., Torgerson P., Martinez-Silveira M.S., Stein C., Abela-Ridder B., Ko A.I. (2015). Global Morbidity and Mortality of Leptospirosis: A Systematic Review. PLoS Negl. Trop. Dis..

[2] Goarant C., Picardeau M., Morand S., McIntyre K.M. (2019). Leptospirosis under the bibliometrics radar: Evidence for a vicious circle of neglect. J. Glob. Health.

[3] Di Bari C., Venkateswaran N., Fastl C., Gabriël S., Grace D., Havelaar A.H., Huntington B., Patterson G.T., Rushton J., Speybroeck N. (2023). The global burden of neglected zoonotic diseases: Current state of evidence. One Health.

[4] Samrot A.V., Sean T.C., Bhavya K.S., Sahithya C.S., Chan-Drasekaran S., Palanisamy R., Robinson E.R., Subbiah S.K., Mok P.L. (2021). Leptospiral Infection, Pathogenesis and Its Diagnosis—A Review. Pathogens.

[5] European Centre for Disease Prevention and Control (2024). Leptospirosis. ECDC. Annual Epidemiological Report for 2022.

[6] Almeida D.S., Paz L.N., de Oliveira D.S., Silva D.N., Ristow P., Hamond C., Costa F., Portela R.W., Estrela-Lima A., Pinna M.H. (2019). Investigation of chronic infection by *Leptospira* spp. in asymptomatic sheep slaughtered in slaughterhouse. PLoS ONE.

[7] Dufour B., Moutou F., Hattenberger A.M., Rodhain F. (2008). Global change: Impact, management, risk approach and health measures-the case of Europe. Rev. Sci. Tech..

[8] Semenza J.C., Menne B. (2009). Climate change and infectious diseases in Europe. Lancet Infect. Dis..

[9] Sohm C., Steiner J., Jöbstl J., Wittek T., Firth C., Steinparzer R., Desvars-Larrive A. (2023). A systematic review on leptospirosis in cattle: A European perspective. One Health.

[10] Loureiro A.P., Lilenbaum W. (2020). Genital bovine leptospirosis: A new look for an old disease. Theriogenology.

[11] Regulation (EU) 2016/429 of the European Parliament and of the Council of 9 March 2016 on Transmissible Animal Diseases and Amending and Repealing Certain Acts in the Area of Animal Health (‘Animal Health Law’). https://eur-lex.europa.eu/legal-content/EN/TXT/PDF/?uri=CELEX:32016R0429.

[12] Ellis W.A. (2015). Animal leptospirosis. Curr. Top. Microbiol. Immunol..

[13] Pedrosa J., Mendes J., Zambrano J., Carvalho-Costa F.A., Di Azevedo M.I.N., Aymée L., Lilenbaum W. (2025). How Is Bovine Genital Leptospirosis Diagnosed Under Field Conditions?. Animals.

[14] Sohm C., Willixhofer D., Fasching E., Waldner K., Deitzer N., Steiner J., Jöbstl J., Schleicher C., Schwarz M., Fuchs R. (2024). First isolation and genotyping of pathogenic *Leptospira* spp. from Austria. Sci. Rep..

[15] Aymée L., Gregg W.R.R., Loureiro A.P., Di Azevedo M.I.N., Pedrosa J.S., Melo J.D.S.L., Carvalho-Costa F.A., de Souza G.N., Lilenbaum W. (2021). Bovine Genital Leptospirosis and reproductive disorders of live subfertile cows under field conditions. Vet. Microbiol..

[16] Aymée L., Mendes J., Lilenbaum W. (2024). Bovine Genital Leptospirosis: An Update of This Important Reproductive Disease. Animals.

[17] Jones A.L. (2024). Sexually Transmitted Diseases of Bulls. Vet. Clin. N. Am. Food Anim. Pract..

[18] O’ Doherty E., Sayers R., O’ Grady L., Shalloo L. (2015). Effect of exposure to *Neospora caninum*, *Salmonella*, and *Leptospira interrogans* serovar Hardjo on the economic performance of Irish dairy herds. J. Dairy Sci..

[19] Carvalho H.G.A.C., Silva D.M., Rodrigues G.R.D., Gameiro A.H., Dos Santos R.F., Raineri C., Lima A.M.C. (2024). Estimation of economic losses due to leptospirosis in dairy cattle. Prev. Vet. Med..

[20] Grégoire F., Bakinahe R., Petitjean T., Boarbi S., Delooz L., Fretin D., Saulmont M., Mori M. (2020). Laboratory Diagnosis of Bovine Abortions Caused by Non-Maintenance Pathogenic *Leptospira* spp.: Necropsy, Serology and Molecular Study Out of a Belgian Experience. Pathogens.

[21] Maiolino S.R., Cortez A., Langoni H., Giuffrida R., Dos Santos J.R., de Nardi Júnior G., Lara G.H.B., Motta R.G., Chacur M.G.M., Monteiro F.M. (2021). Sperm viability, serological, molecular, and modified seminal plasma agglutination tests in the diagnosis of Leptospira in the semen and serum of bovine bulls. Braz. J. Microbiol..

[22] World Organization for Animal Health (WOAH) (2023). Manual of Diagnostic Tests and Vaccines for Terrestrial Animals.

[23] Mazzotta E., Bellinati L., Bertasio C., Boniotti M.B., Lucchese L., Ceglie L., Martignago F., Leopardi S., Natale A. (2023). Synanthropic and Wild Animals as Sentinels of Zoonotic Agents: A Study of *Leptospira* Genotypes Circulating in Northeastern Italy. Int. J. Environ. Res. Public Health.

[24] Smythe L.D., Smith I.L., Smith G.A., Dohnt M.F., Symonds M.L., Barnett L.J., McKay D.B. (2002). A quantitative PCR (TaqMan) assay for pathogenic *Leptospira* spp.. BMC Infect. Dis..

[25] Bedir O., Kilic A., Atabek E., Kuskucu A.M., Turhan V., Basustaoglu A.C. (2010). Simultaneous detection and differentiation of pathogenic and nonpathogenic *Leptospira* spp. by multiplex real-time PCR (TaqMan) assay. Pol. J. Microbiol..

[26] Stoddard R.A., Gee J.E., Wilkins P.P., McCaustland K., Hoffmaster A.R. (2009). Detection of pathogenic *Leptospira* spp. through TaqMan polymerase chain reaction targeting the LipL32 gene. Diagn. Microbiol. Infect. Dis..

[27] Boonsilp S., Thaipadungpanit J., Amornchai P., Wuthiekanun V., Bailey M.S., Holden M.T.G., Zhang C., Jiang X., Koizumi N., Taylor K. (2013). A Single Multilocus Sequence Typing (MLST) Scheme for Seven Pathogenic Leptospira Species. PLoS Negl. Trop. Dis..

[28] Bertasio C., Papetti A., Scaltriti E., Tagliabue S., D’Incau M., Boniotti M.B. (2020). Serological Survey and Molecular Typing Reveal New *Leptospira* Serogroup Pomona Strains among Pigs of Northern Italy. Pathogens.

[29] Recordati C., Radaelli E., Simpson K.W., Scanziani E. (2008). A simple method for the production of bacterial controls for immunohistochemistry and fluorescent in situ hybridization. J. Mol. Histol..

[30] Moinet M., Wilkinson D.A., Aberdein D., Russell J.C., Vallée E., Collins-Emerson J.M., Heuer C., Benschop J. (2021). Of Mice, Cattle, and Men: A Review of the Eco-Epidemiology of *Leptospira borgpetersenii* Serovar Ballum. Trop. Med. Infect. Dis..

[31] Delooz L., Czaplicki G., Gregoire F., Dal Pozzo F., Pez F., Kodjo A., Saegerman C. (2018). Serogroups and genotypes of *Leptospira* spp. strains from bovine aborted foetuses. Transbound. Emerg. Dis..

[32] Aliberti A., Blanda V., Di Marco Lo Presti V., Macaluso G., Galluzzo P., Bertasio C., Sciacca C., Arcuri F., D’Agostino R., Ippolito D. (2022). *Leptospira interrogans* Serogroup Pomona in a Dairy Cattle Farm in a Multi-Host Zootechnical System. Vet. Sci..

[33] Yupiana Y., Wilson P.R., Weston J.F., Vallée E., Collins-Emerson J.M., Benschop J., Scotland T., Heuer C. (2019). Epidemiological investigation of *Leptospira* spp. in a dairy farming enterprise after the occurrence of three human leptospirosis cases. Zoonoses Public Health.

[34] Rocha B.R., Martins G., Lilenbaum W. (2020). An historical view of the experimental leptospiral infection in ruminants. Comp. Immunol. Microbiol. Infect. Dis..

[35] Molinari P.C.C., Nally J.E., Bromfield J.J. (2021). Bovine endometrial cells do not mount an inflammatory response to *Leptospira*. Reprod. Fertil..

[36] Thiermann A.B. (1982). Experimental leptospiral infections in pregnant cattle with organisms of the Hebdomadis serogroup. Am. J. Vet. Res..

[37] Nally J.E., Ahmed A.A.A., Putz E.J., Palmquist D.E., Goris M.G.A. (2020). Comparison of Real-Time PCR, Bacteriologic Culture and Fluorescent Antibody Test for the Detection of *Leptospira borgpetersenii* in Urine of Naturally Infected Cattle. Vet. Sci..

[38] Wagenaar J., Zuerner R.L., Alt D., Bolin C.A. (2000). Comparison of polymerase chain reaction assays with bacteriologic culture, immunofluorescence, and nucleic acid hybridization for detection of *Leptospira borgpetersenii* serovar hardjo in urine of cattle. Am. J. Vet. Res..

[39] Zarantonelli L., Suanes A., Meny P., Buroni F., Nieves C., Salaberry X., Briano C., Ashfield N., Da Silva Silveira C., Dutra F. (2018). Isolation of pathogenic *Leptospira* strains from naturally infected cattle in Uruguay reveals high serovar diversity, and uncovers a relevant risk for human leptospirosis. PLoS Negl. Trop. Dis..

[40] Hernández-Rodríguez P., Díaz C.A., Dalmau E.A., Quintero G.M. (2011). A comparison between polymerase chain reaction (PCR) and traditional techniques for the diagnosis of leptospirosis in bovines. J. Microbiol. Methods.

[41] Hamond C., LeCount K., Putz E.J., Bayles D.O., Camp P., Goris M.G.A., van der Linden H., Stone N.E., Schlater L.K., Sahl J.W. (2022). Bovine Leptospirosis Due to Persistent Renal Carriage of *Leptospira borgpetersenii* Serovar Tarassovi. Front. Vet. Sci..

[42] Monti G., Montes V., Tortosa P., Tejeda C., Salgado M. (2023). Urine shedding patterns of pathogenic *Leptospira* spp. in dairy cows. Vet. Res..

[43] (2016). Jubb, Kennedy & Palmer’s Pathology of Domestic Animals.

[44] Bonaparte A., Page C., Beeler E. (2018). Orchitis and balanoposthitis in a dog with *Leptospira interrogans* serovar Canicola in Southern California. Vet. Rec. Case Rep..

